# Generating Seamless Three-Dimensional Maps by Integrating Low-Cost Unmanned Aerial Vehicle Imagery and Mobile Mapping System Data

**DOI:** 10.3390/s25030822

**Published:** 2025-01-30

**Authors:** Mohammad Gholami Farkoushi, Seunghwan Hong, Hong-Gyoo Sohn

**Affiliations:** 1 School of Civil and Environmental Engineering, Yonsei University, Seodaemun-gu, Seoul 03722, Republic of Korea; mohamad_gholami@yonsei.ac.kr; 2 Kakao Mobility, 152, Pangyoyeok-ro, Bundang-gu, Seongnam-si 13529, Republic of Korea; hongseunghwan0@gmail.com

**Keywords:** UAV, MMS, point cloud

## Abstract

This study introduces a new framework for combining calibrated mobile mapping system (MMS) data and low-cost unmanned aerial vehicle (UAV) images to generate seamless, high-fidelity 3D urban maps. This approach addresses the limitations of single-source mapping, such as occlusions in aerial top views and insufficient vertical detail in ground-level data, by utilizing the complementary strengths of the two technologies. The proposed approach combines cloth simulation filtering for ground point extraction from MMS data with deep-learning-based segmentation (U²-Net) for feature extraction from UAV images. Street-view MMS images are projected onto a top-down viewpoint using inverse perspective mapping to align diverse datasets, and precise cross-view alignment is achieved using the LightGlue technique. The spatial accuracy of the 3D model was improved by integrating the matched features as ground control points into a structure from the motion pipeline. Validation using data from the campus of Yonsei University and the nearby urban area of Yeonhui-dong yielded notable accuracy gains and a root mean square error of 0.131 m. Geospatial analysis, infrastructure monitoring, and urban planning can benefit from this flexible and scalable method, which enhances 3D urban mapping capabilities.

## 1. Introduction

The requirement for high-resolution and comprehensive three-dimensional (3D) spatial data has expanded considerably recently owing to the current needs in specific domains, including autonomous navigation, disaster management, and urban planning [1,2,3]. Accurate 3D mapping is an essential component of decision-support systems used in environmental management, infrastructure monitoring, and smart city development [4,5,6]. Although traditional 3D data collection techniques can cover wide regions, they typically lack the resolution and accuracy required for dense urban environments where tightly packed structures and complicated topographies create major occlusions [7,8,9].

Recently, unmanned aerial vehicles (UAVs) and mobile mapping systems (MMSs) have been recognized as transformative technologies for high-resolution 3D mapping, each offering unique advantages. UAVs can capture georeferenced images from an aerial perspective in a flexible and cost-efficient manner [10]. Their wide coverage area is particularly well suited to urban environments [11,12]. However, they often cause occlusions because of their single view, top-down capture, and limited vertical information [13,14]. Conversely, MMS platforms equipped with light detection and ranging (LiDAR) sensors, cameras, and global navigation satellite systems and inertial navigation systems (GNSSs/INSs) generate dense point clouds from ground-level views, enabling detailed mapping of vertical structures, but lack above-surface coverage for complete 3D representations [15,16]. To address these limitations, data fusion techniques have recently been explored to combine the complementary strengths of UAV and MMS systems, enabling the creation of seamless and comprehensive 3D models [17,18].

Real-time kinematic (RTK) and post-processed kinematic (PPK) solutions have become prevalent for UAV-based georeferencing, achieving centimeter-level accuracy [19,20,21]. However, these methods increase operational costs and complexity. Alternatively, the indirect approach of using ground control points (GCPs) ensures high geolocation accuracy if the quality and distribution of GCPs are properly managed. This study demonstrates the feasibility of achieving precise integration between low-cost UAV and MMS datasets without relying on RTK/PPK solutions, which makes the proposed method more cost-effective and adaptable to diverse applications.

Recent studies have explored various data-fusion approaches for integrating UAV and MMS datasets to achieve high-accuracy 3D mapping, particularly in urban and complex environments [22,23]. Multiple studies have adopted innovative methods to overcome the challenges related to data perspective, scale, and resolution. One approach involves the use of MMS point clouds as indirect GCPs to improve UAV-based photogrammetry. For instance, a strategy has been proposed that utilizes feature points from MMS data, such as building façades and road markings, as GCPs to enhance the geolocation accuracy of low-cost UAV photogrammetric models [24]. This method improved considerably the vertical geolocation accuracy of UAV data by diversely distributing MMS-derived GCPs along the vertical axis, thereby reducing the positional error to less than 16 cm in all directions. This indirect georeferencing approach streamlines the traditional labor-intensive GCP acquisition process by substituting MMS data with spatially distributed GCPs, creating an efficient framework for high-resolution 3D urban reconstruction.

The efficacy of UAV-LiDAR systems for generating accurate 3D point clouds in challenging coastal environments, while emphasizing the cost-effectiveness of UAV-based methodologies, has been demonstrated [25]. Similarly, clustering-based registration techniques have been showcased to enhance the alignment of UAV and terrestrial LiDAR point clouds, facilitating robust forest structure analyses [26]. Furthermore, the potential of automated ground control point extraction from UAV imagery and mobile mapping systems for streamlined aerial triangulation has been highlighted, reinforcing the utility of such integrations for urban mapping [27].

Another common fusion method involves feature-based matching, in which features in both UAV images and MMS point clouds are detected and aligned using algorithms, such as scale-invariant feature transforms (SIFTs) and speeded-up robust features (SURFs) [28,29]. However, these approaches often fail in real-world scenarios with large differences in viewpoint and scale, resulting in incomplete or inaccurate data alignment [30]. Some studies have also relied on manually selected GCPs to align the UAV and MMS data by using a common coordinate system, although this process can be resource-intensive for large or dynamic datasets [31].

Structure-from-motion (SfM) [32] and iterative closest point (ICP) [33] are commonly used in data fusion workflows. SfM is typically employed to build UAV-based 3D models that are then aligned with MMS point clouds using ICP algorithms to refine spatial positioning. However, scale inconsistencies between UAV and MMS data often necessitate additional refinement for effective integration [34,35]. In another study [36], an integrated approach was proposed that combined UAV-based photogrammetry with terrestrial laser scanning (TLS) to map open-pit mines. Their methodology incorporated a bundle adjustment process using TLS-derived GCPs along with UAV imagery to improve the geo-referencing accuracy in high-risk areas where traditional GPS-based GCP collection is challenging. This approach achieved decimeter-level accuracy for digital surface models and provided a robust solution for 3D modeling in mountainous and inaccessible regions [37,38].

Using cutting-edge methods for data fusion, feature extraction, and alignment, this study addresses these issues by proposing an effective and expandable framework for producing seamless, high-fidelity 3D urban maps. The use of cloth simulation filtering (CSF) to extract ground points from MMS datasets and U²-Net, a deep-learning model for segmenting road features in UAV images, are two important developments. Additionally, the methodology uses LightGlue, a deep-learning-based feature-matching algorithm, to accomplish precise cross-view alignment and introduces inverse perspective mapping (IPM) to align MMS street-view images with UAV top-down data. By integrating these matched features as GCPs into an SfM pipeline, the spatial accuracy of the final 3D model is greatly improved, and both the exterior and interior orientation parameters are refined. By combining these advanced techniques, the proposed framework delivers high-resolution, spatially accurate 3D urban models.

Data from the nearby Yeonhui-dong neighborhood and the Sinchon campus of Yonsei University in Seoul, South Korea, were utilized to validate the framework. Owing to their complex building structures, diverse topography, and heavily populated infrastructure, these regions reflect a variety of metropolitan environments. With a root mean square error (RMSE) of 0.131 m, the results yielded notable gains in spatial alignment, surpassing prior standards and offering comprehensive urban mapping appropriate for a range of geospatial applications.

The remainder of this paper is organized as follows: Section 2.1 describes the study area and data collection methods, detailing the specifications of the UAV and MMS platforms used. Section 2.2 presents the methodology, including data preprocessing techniques, feature extraction, and matching methods, such as LightGlue and U²-Net. Section 3 discusses the results of the data fusion process, focusing on spatial accuracy and feature completeness. Finally, Section 4 explores the broader implications of the findings and proposes directions for future research on 3D urban mapping.

## 2. Materials and Methods

### 2.1. Study Site

**Figure 1:**
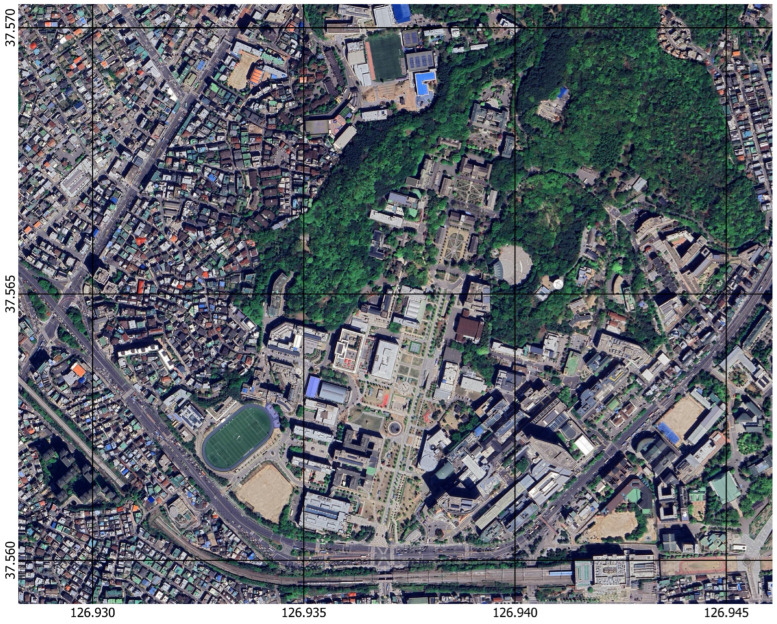
Geographical overview of Yonsei University’s Sinchon campus and the Yeonhui-dong.

Data from Seoul, South Korea’s Yeonhui-dong neighborhood, and the Sinchon campus of Yonsei University were used in this study. The geographical layout of the study area is depicted in Figure 1, which also highlights the major landmarks and areas of interest examined in this study. This area was selected because of its varied urban features, which make it the perfect place for testing and validating the proposed 3D mapping framework. The research site is representative of a typical dense urban setting as it covers an area of approximately 1.28 km² and includes various building types, road networks, and topographical features.

While Yonsei University’s Sinchon campus is distinguished by modern academic buildings surrounded by natural areas, Yeonhui-dong district is home to a densely populated residential and commercial zone with small streets and varying elevations. This combination of structured and unstructured environments introduces major challenges for 3D mapping, including (1) occlusions caused by high-rise buildings and densely packed infrastructure, (2) varied terrain with elevation changes requiring robust ground feature extraction, and (3) complex road networks that test the ability of feature-matching algorithms to achieve accurate alignment across datasets.

Owing to these urban complications, an effective method that can seamlessly combine ground-level and aerial data is required. The chosen site not only reflects real-world mapping conditions but also provides diverse features that emphasize the capabilities of the proposed framework. For example, different road layouts and building façades provide opportunities to assess how well the LightGlue algorithm performs in feature matching and how the U²Net model performs in feature segmentation.

These urban complexities necessitate a robust methodology that is capable of seamlessly integrating aerial and ground-level data. The chosen site not only reflects real-world mapping conditions but also provides diverse features that stress the capabilities of the proposed framework. For instance, various building façades and road layouts offer opportunities to evaluate the performance of the U²-Net model in feature segmentation and the LightGlue algorithm in feature matching.

### 2.2. Data Collection

#### 2.2.1. UAV Data Acquisition

A WingtraOne UAV was used to collect high-resolution aerial images of the study area. Flying was conducted at a uniform altitude of 100 m from the ground to achieve an optimal balance between image resolution and coverage. A total of 4000 images with a ground sampling distance of 2 cm were captured, allowing a full and detailed geographic investigation of both locations. The selected UAV was WingtraOne, Wingtra, Zurich, Switzerland, based on the complex features of the UAV, because it had accurate image acquisition, long endurance, and was quite adaptive to diverse environments. The capability of this UAV has proven very useful in operation in urban and congested areas by enabling the deployment and recovery of the UAV in places that have inadequate ground space for conventional runway facilities. The high-resolution imagery from the payload of the WingtraOne further enhances surface analysis to extensive levels, aiding in generating accurate and reliable data outputs. Table 1 lists the specifications of WingtraOne UAV.

WingtraOne’s high-quality payload, the Sony RX1R II, captures extremely detailed red–green–blue (RGB) images. WingtraOne carries a top-of-the-line payload called the Sony RX1R II, which captures highly detailed RGB images. This impressive endurance, possibly as long as 59 min per flight, allows us to fly over considerable distances without requiring excessive recharge, thus optimizing efficiency. The extended endurance allowed us to fly uninterrupted sessions of thorough data acquisition, adding to the high-quality dataset needed for subsequent analyses. The capabilities of WingtraOne were instrumental in achieving precise data collection over both the Yonsei campus and Yeonhui-dong, setting a solid foundation for accurate geospatial analysis.

#### 2.2.2. MMS Data Acquisition

To support our UAV imagery for full urban mapping and analysis, we gathered high-density point cloud data and street-view images using a Pegasus Leica MMS. This acquisition focused on the Yonsei University campus and Yeonhui-dong area and collected fine ground details from street level. Using the Pegasus Leica system for approximately 1 h, we accumulated a large dataset with approximately 480 million points from a road distance of 3.7 km. This dataset covers not only structural details but also important geographical features, allowing for a comprehensive and integrated perspective of the environment.

Each of these points in the cloud is enriched with RGB color data, intensity information, and GNSS coordinates; hence, it is a very detailed and accurate digital representation of the physical environment. The RGB color value allows for the true color presentation of the object, enhancing the accuracy of 3D models, and making the point cloud ready for visual presentation. Intensity values provide additional information on surface quality and enable subtle inquiries across diverse materials or textures. The GNSS data ensure that every point is geolocated with precision, thereby meeting the requirements of any given task. Table 2 lists the key specifications of the Pegasus Leica mobile mapping system.

The Pegasus Leica system was developed specifically for mobile mapping, excelling in urban and constrained areas, where mobility and quickness are critical. Its high capture rate and precise optics enable the collection of extremely detailed spatial data while navigating complex streets. This mobile mapping device is outfitted with cutting-edge sensors and processing technology, allowing the real-time recording of large amounts of point data without losing detail or accuracy. We calibrated these datasets using the algorithm proposed in [39], which improved the accuracy, resulting in a post-calibration accuracy likely in the range of 10–12 mm.

**Figure 2:**
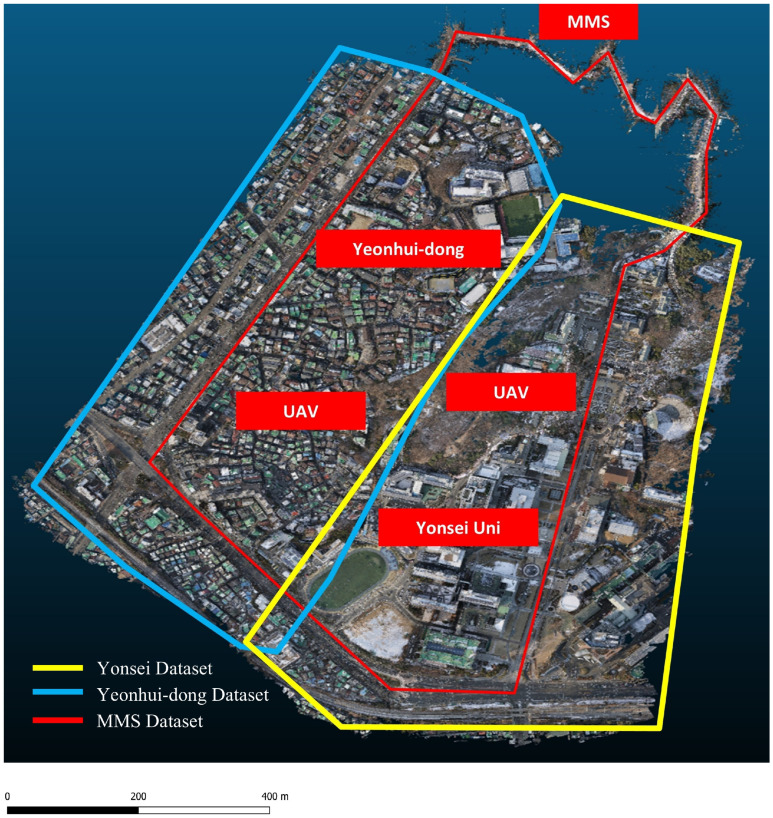
Visual representations of data collected using UAV and MMS.

Merging the Pegasus Leica system’s ground-level data with airborne data from the WingtraOne UAV provided a coupled geographical dataset that combined representatives from high-altitude and street-level perspectives. This broadens our knowledge of urban settings, while simultaneously enabling a multiscale study of building facades, ground surfaces, and infrastructure. The result is a combined dataset capable of full 3D modeling and urban analysis, which provides essential information for a myriad of geospatial and planning applications. Figure 2 shows the data acquired over the study region and mapped onto a real-world environment.

### 2.3. Methods

In this study, low-cost UAV images were combined with calibrated MMS point cloud data to generate 3D point clouds. The procedure begins by processing UAV images using an artificial intelligence (AI) approach to extract road data. An MMS-calibrated point cloud was used to extract road information using the CFS approach. Roads were extracted from the MMS dataset and converted into top-view images. IPM was applied to MMS street-view images to convert them into top-view perspectives, thereby enabling effective feature matching between UAV imagery and the MMS dataset.

Feature matching between the UAV and MMS top-view images was performed to match the respective road features. This alignment improved the uniformity of the extracted road features. Furthermore, the matched features from the two datasets served as GCPs to improve the estimate of the exterior orientation parameters (EOPs) and interior orientation parameters (IOPs) during the SfM approach. By incorporating these matched features into the SfM method, the accuracy of the ensuing 3D point cloud reconstruction improved dramatically.

To facilitate this integration, UAV road features were segmented using U²-Net, while the MMS dataset was processed using cloth simulation filtering (CSF) to isolate ground-level details. MMS street-view images were transformed into a top-down view using inverse perspective mapping (IPM) to ensure consistency in perspective between the UAV and MMS datasets.

LightGlue, a deep-learning-based feature-matching algorithm, was used for precise alignment of UAV and MMS data, overcoming challenges of large-scale differences and viewpoint variations. The matched features were incorporated as GCPs in the structure-from-motion (SfM) pipeline, refining both EOPs and IOPs and improving spatial accuracy. The MMS point cloud was treated as the reference during iterative closest point (ICP) registration to align the UAV-derived point cloud. This process minimized spatial distortions, ensuring seamless integration of the two datasets.

**Figure 3:**
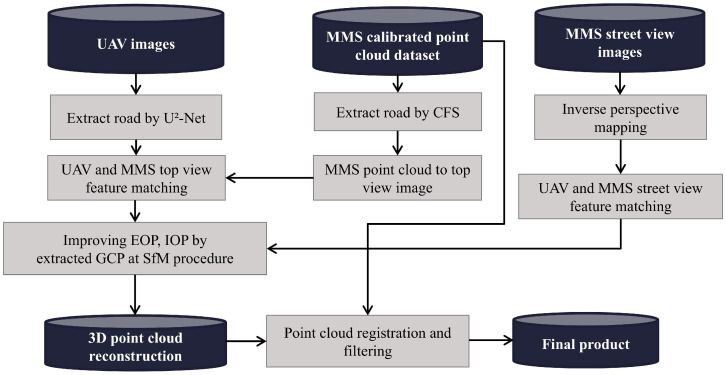
Flowchart depicting the proposed approach.

Subsequently, a 3D point cloud was reconstructed from the UAV images using enhanced orientation parameters. The MMS point cloud was then registered and filtered against the reconstructed UAV point cloud to ensure proper alignment and noise reduction. Following the registration and filtering processes, a refined 3D point cloud model was created and used as the final product for analysis. Figure 3 illustrates the flow of this research.

#### 2.3.1. Road Extraction from UAV Images

The extraction of roads and streets from the UAV images was performed using an AI-based method. In this regard, an AI model was specially trained to identify and segment roads using aerial imagery. Subsequently, the preprocessing of the UAV images was refined to prepare them for application in the AI model. This resulted in a set of feature-based road masks for roads in the UAV images. These extracted road features are important for further processing because they provide the basis for feature matching with the MMS dataset.

In this study, we adopted U^2^-Net [40], which was developed for salient object detection using deep-learning models, to conduct road detection from UAV imagery. The architecture of U^2^-Net applies an improved structure from the traditional U-Net by embedding ReSidual U-block (RSU) blocks within an encoder–decoder framework with a large “U” shape. It can capture both global contextual information and fine-grained local details owing to its multilevel framework, and it is particularly suitable for complex segmentation tasks such as road detection.

RSU blocks are crucial for the success of U^2^-Net. Each block may be viewed as a “mini-U-Net” in the network, extracting features across multiple scales. These U-shaped pathways realize hierarchical feature learning in the U-Net. U^2^-Net captures broad contextual information by progressive downsampling in the encoder phase and restores spatial details by upsampling and combining multiscale features from earlier layers in the decoder phase, as shown in Equation (1):

(1) $$F_{\mathrm{out}}=f_{\mathrm{upsample}}\left(f_{\mathrm{conv}}\left(f_{\mathrm{downsample}}\left(F_{\mathrm{in}}\right)\right)+F_{\mathrm{skip}}\right)$$

where $F_{\mathrm{in}}$ is the input feature map, $f_{\mathrm{downsample}}$ represents the downsampling operation, $f_{\mathrm{conv}}$ is the convolutional operation, $f_{\mathrm{upsample}}$ is the upsampling operation, and $F_{\mathrm{skip}}$ denotes the skip connection.

The training process minimizes a combined loss function to balance pixel-wise accuracy and segmentation quality, defined as Equation (2):

(2) $$L_{\mathrm{total}}=\alpha L_{\mathrm{CE}}+\beta L_{\mathrm{IoU}}$$

where $L_{\mathrm{CE}}$ is the cross-entropy loss, $L_{\mathrm{IoU}}$ is the intersection-over-union loss, and α and β are weighting factors for balancing the two components.

The key capabilities of U^2^-Net are based on the ability of the network to focus selectively on salient regions, enabling it to concentrate on road structures while retaining critical edges and information. This not only reduces background interference but also enhances the visibility of segmented road regions. Therefore, U^2^-Net is well-suited for UAV-based road detection tasks.

We used a dataset composed of 320 UAV images for training and validation; each image was manually annotated to provide accurate ground-truth labels for road segmentation. It was ensured that the manual extraction of ground-truth data produced high-quality annotations that can accurately represent the boundaries along with the structural complexity of roads. Models such as U^2^-Net require a high-quality ground truth, which relies on precise edge detection and context retention. This enables U^2^-Net to learn from these data the ability to perceive road features with high accuracy in a variety of scenarios, including those where segmentation may be affected by light conditions, terrain, or salient background objects.

The training of a U^2^-Net model on manually annotated UAV images can result in high performance in road detection tasks. The model was trained on the system using an NVIDIA GeForce RTX 2080 Ti graphics processing unit that provided the necessary computing power for deep learning and high-resolution UAV images. The performance of the proposed model was assessed using several key metrics. The overall accuracy, which was 96.38%, refers to the percentage of properly classified pixels as road or nonroad within an image, thus providing a general view of model performance. The overall accuracy is defined as Equation (3):

(3) $$\mathrm{Accuracy}=\frac{TP+TN}{TP+TN+FP+FN}$$

where TP, TN, FP, and FN represent true positives, true negatives, false positives, and false negatives, respectively.

However, because road segmentation tasks are usually highly imbalanced, the accuracy is not sufficient to capture the effectiveness of the model. The *F*1-score, which balances precision and recall, was 92.92%. The *F*1-score is computed as Equation (4):

(4) $$F1=2\cdot \frac{\mathrm{Precision}\cdot \mathrm{Recall}}{\mathrm{Precision}+\mathrm{Recall}}$$

where

(5) $$\mathrm{Precision}=\frac{TP}{TP+FP},\;\mathrm{Recall}=\frac{TP}{TP+FN}.$$

**Figure 4:**
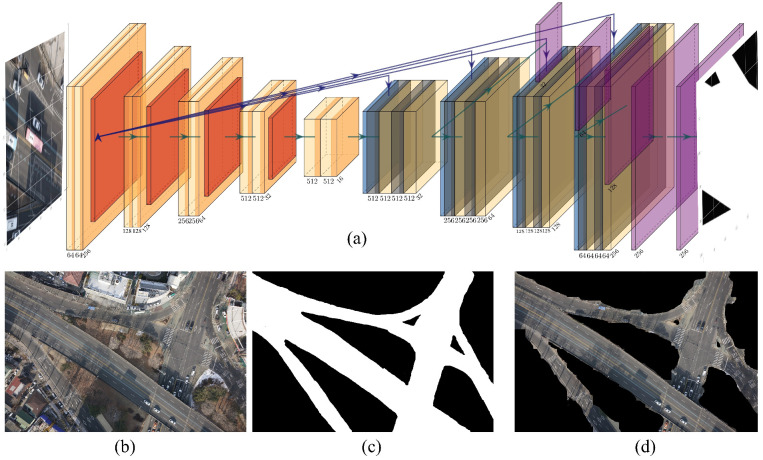
(**a**) Model structure based on U2net, (**b**), UAV image, (**c**) ground truth, (**d**) result of road segmentation.

A high *F*1-score signifies that the model is both accurate and sufficiently complete to mark regions as roads. Together, these metrics provide evidence of the robustness and precision of U^2^-Net for detecting roads using UAV imagery. The proposed U^2^-Net architecture, combined with careful manual data annotation, has proven effective in ensuring high-quality road segmentation, which is crucial in UAV-based applications where accurate mapping, navigation, and infrastructure monitoring are highly stressed in complex environments. Figure 4 presents the U-Net-based structure and road segmentation results from the UAV images processed using this model.

#### 2.3.2. MMS Point Cloud Dataset Processing

A CSF [41] was used to separate the ground points from the point cloud data by filtering buildings, trees, and all other aboveground features from the ground surface. Based on the physics of cloth, these algorithms simulate a virtual “cloth” draped over a landscape. CSF identifies ground points by deforming towards low-lying regions, while leaving elevated points above the cloth. For this purpose, the CSF algorithm begins with the raised starting position of the cloth above the highest points in the point cloud. Subsequently, simulated gravity was applied, which caused the cloth to fall and sit on the ground. In this case, the equation representing gravity is given by Equation (6):

(6) $$\vec{F}=m\cdot \vec{a}$$

where $\vec{F}$ is the gravitational force acting on each clothing vertex, and *m* and $\vec{a}$ are the mass of each vertex and gravitational acceleration, respectively. Accordingly, pulling every vertex downward toward the ground updates its position iteratively to be closer to the ground points (while maintaining points higher than the cloth such as buildings or trees). The following equation expresses the time step to make the cloth physically go down toward the floor, as shown in Equation (7):

(7) $${\vec{p}}_{\mathrm{new}}={\vec{p}}_{\mathrm{current}}+\vec{v}\Delta t+\frac{1}{2}\vec{a}\Delta t^{2}$$

where ${\vec{p}}_{\mathrm{current}}$ is the current position of this vertex, $\vec{v}$ is the initial velocity, and Δt is the time step to make the cloth physically go down towards the floor.

The cloth was constrained to be stiff to retain its realistic shape. These stiffness constraints prevented excessive bending, thereby allowing for a smooth and continuous draping effect over the ground. This was achieved by implementing spring-like forces between the neighborhood vertices of the cloth, defined by Equation (8):

(8) $${\vec{F}}_{\mathrm{spring}}=-k\left(\vec{d}-{\vec{d}}_{\mathrm{rest}}\right)$$

where *k* is the constant of stiffness, $\vec{d}$ is the current distance between the vertices, and ${\vec{d}}_{\mathrm{rest}}$ is the ideal spacing. This force keeps the vertices close to each other, mimics the tensile strength of the fabric, and helps the cloth lay naturally over ground points rather than deeply penetrating them.

**Figure 5:**
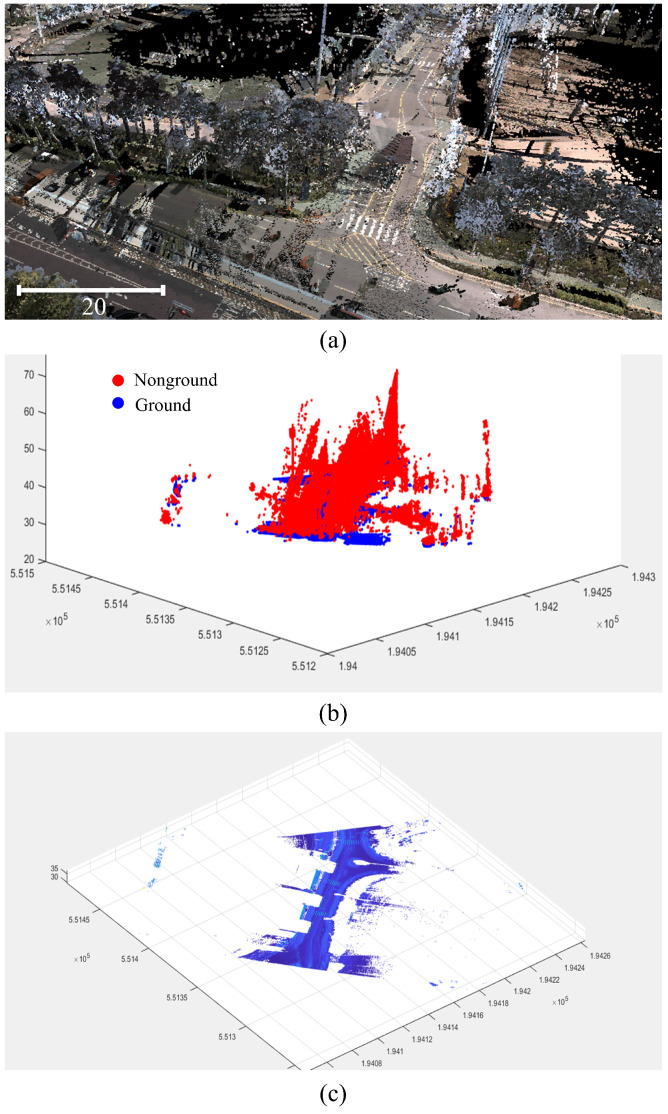
CFS results. (**a**) MMS point cloud, (**b**) segmented point cloud, and (**c**) ground point cloud.

After the cloth reached a resting state, the points touching it were classified as ground, and the points above it were marked as nonground. The optimization of ground surface filtering in our urban environment was achieved by tuning parameters such as cloth stiffness, grid density, and number of iterations. Figure 5 shows the segmented ground point clouds obtained using the CFS.

#### 2.3.3. MMS Point Cloud to Top-View Image

**Figure 6:**
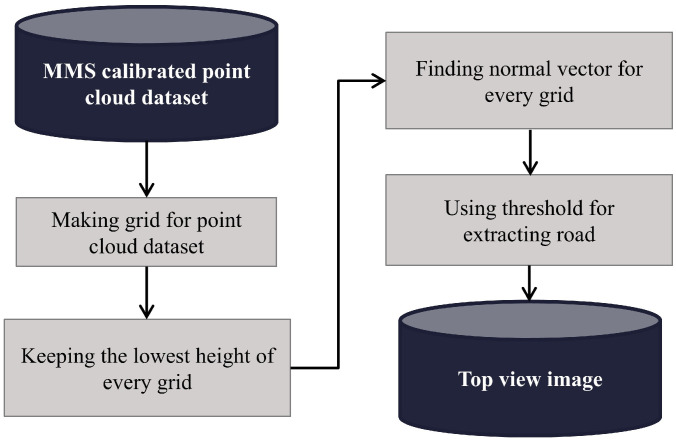
Point cloud to top-view image conversion flowchart.

Our algorithm effectively projects a 3D point cloud onto a 2D top-view intensity image using both the spatial and intensity information from the point cloud. It can operate on point cloud data stored in several formats, perform aggregation and statistical analysis based on grids, and visualize top-down intensities along with other properties. First, the algorithm loads the data from a point cloud and extracts the essential attributes, including the X, Y, and Z spatial coordinates, intensity values, GNSS timestamps, and RGB color channels. Structuring data into matrices enables systematic aggregation within a defined spatial grid and further analysis. Figure 6 presents a flowchart detailing the process of transforming a 3D point cloud into a top-view 2D image.

This grid was produced by dividing the extent of the point cloud into cells in the X and Y directions, given a specified gridding size. In this study, the grid size was set to three units. The boundary of the grid was defined based on the minimum and maximum values of X and Y, whereas the spatial resolution of each cell was controlled by the size of the grid. Thus, we initiated the matrices of density, intensity, and other statistical characteristics of the Z-dimension along with the RGB values and intensity levels across the grid.

First, we prepared the grid and assigned each point in the point cloud to its corresponding grid cell. Subsequently, we computed the properties per cell, such as the density (the count of points per cell), cumulative height, intensity values, and RGB components. Metrics such as the mean, standard deviation, variance, and minimum and maximum can be obtained by averaging, normalizing, or otherwise processing the accumulated values in each cell.

Mathematically, let G(x,y) represent a grid cell at position (x,y) in the 2D plane. The density D(x,y), average height $\bar{Z}\left(x,y\right)$, and normalized intensity I(x,y) are calculated as Equations (9)–(11):

(9) $$D\left(x,y\right)=\sum_{i=1}^{N}\delta \left(x_{i},y_{i}\right),$$

(10) $$\bar{Z}\left(x,y\right)=\frac{1}{D(x,y)}\sum_{i=1}^{N}Z_{i}\cdot \delta \left(x_{i},y_{i}\right),$$

(11) $$I\left(x,y\right)=\frac{1}{D(x,y)}\sum_{i=1}^{N}I_{i}\cdot \delta \left(x_{i},y_{i}\right)$$

where $\delta (x_{i},y_{i})=1$ if the point $\left(x_{i},y_{i}\right)$ lies within the grid cell G(x,y), and 0 otherwise. Here, *N* represents the total number of points in the point cloud.

Additionally, Delaunay triangulation is applied to compute normal vectors for triangular faces in the point cloud. Points with normal vectors forming an angle less than $5^{\circ }$ with the vertical axis are retained to emphasize planar surfaces, such as roads or flat areas, in the top-view intensity image. These planar regions are visualized separately to highlight structural features in the point cloud.

These averaged values were normalized, and a color map was applied to create a generated 2D intensity image representing the top-down intensity distribution in a human-readable format. The intensity map was then normalized and rescaled for storage as an image. The intensity values were mapped to grayscale. In addition, other 2D maps, such as density, height, and intensity variance maps, can be used to visualize other characteristics of the dataset. These maps represent spatial patterns in the point cloud and enable derivation of the distribution and properties of points in the viewed area.

**Figure 7:**
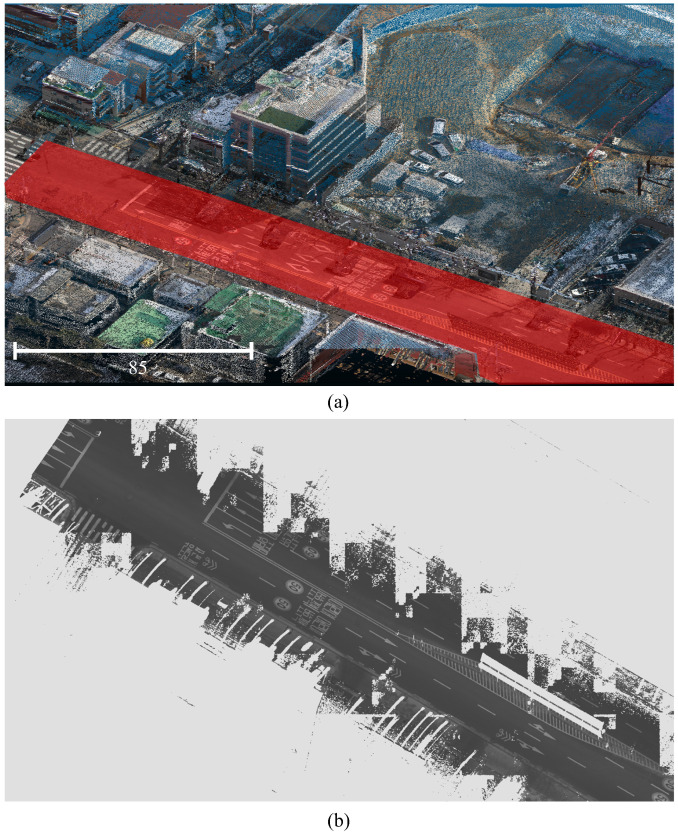
(**a**) Point cloud and (**b**) top-view 2D image from the 3D point cloud.

The 3D point cloud onto a 2D top-view algorithm involves the detection of road-like structures or planarities from a point cloud, for which Delaunay triangulation is applied to calculate the normal vectors at every triangular face. We filtered the points based on the angles created by these normals to identify the regions with specific planar characteristics. For example, the angle between the estimated normal vectors and the z-axis can be used to identify points belonging to nearly horizontal or vertical surfaces. According to these thresholds, the filtering procedure characterizes normals with angles less than a particular threshold as flat or road-like areas, which are visualized in separate 3D plots with respect to the identified planar surfaces. Figure 7 shows the results obtained by converting a 3D point cloud into a 2D image.

#### 2.3.4. Conversion of MMS Street-View to Top-Down View Using IPM

IPM [42] is a geometric transformation technique that projects an image acquired from an oblique or street view onto a bird’s-eye top-down view. By reprojecting the image pixels to a parallel ground plane, IPM removes the perspective distortions that occur when images are acquired from non-orthogonal angles. This is a prerequisite transformation when fusing MMS street-view data with UAV top-down imagery to obtain a consistent overhead projection, which further facilitates the process of accurate feature matching and multiperspective integration.

IPM transformation defines a set of real-world, ground-plane coordinates onto which a street-level image is mapped. The transformation can be modeled using a homography matrix that establishes a projective relationship between the 3D world coordinates and 2D image plane coordinates by considering the ground surface as a flat plane.

Let P=(X,Y,Z) represent the coordinates of a point in real-world 3D space. The corresponding 2D pixel coordinates p=(x,y) in the image plane can be derived using the camera projection matrix. The homography matrix *H* maps points from the image plane to the real-world ground plane and is defined as defined by Equation (12):

(12) $$p^{\prime }=Hp$$

where $p^{\prime }$ is the transformed point on the top-down view, *H* is the homography matrix, and p=(x,y,1) represents the homogeneous coordinates of a point in the original image.

The homography matrix *H* can be constructed as in Equation (13):

(13) $$H=K\cdot \left[R|T\right]$$

where *K* is the camera intrinsic matrix, which includes the focal lengths $f_{x}$, $f_{y}$ and principal points $c_{x}$, $c_{y}$ and is expressed as shown in Equation (14):

(14) $$K=\left[\begin{array}{ccc} f_{x} & 0 & c_{x}\\ 0 & f_{y} & c_{y}\\ 0 & 0 & 1 \end{array}\right]$$

*R* is the rotation matrix that aligns the camera view with the ground plane, and *T* is the translation vector that shifts the origin to the desired ground plane.

To compute the inverse perspective mapping, we assumed a flat ground plane at a fixed height (for example Z=0) in the real world. Under this assumption, we can express the mapping as shown in Equation (15):

(15) $$\left[\begin{array}{c} x^{\prime }\\ y^{\prime }\\ 1 \end{array}\right]=H\cdot \left[\begin{array}{c} X\\ Y\\ 1 \end{array}\right]$$

This projection redefines the (X,Y) coordinates on the ground plane to the image plane’s corresponding pixel coordinates, allowing us to “flatten” the MMS view into a top-down orientation.

For a pixel at (x,y) in the original MMS image, the ground-plane coordinates (X,Y) can be retrieved by computing the inverse of the homography matrix *H*, represented in Equation (16):

(16) $$\left[\begin{array}{c} X\\ Y\\ 1 \end{array}\right]=H^{-1}\cdot \left[\begin{array}{c} x\\ y\\ 1 \end{array}\right]$$

This inverse mapping aligns the transformed MMS street view with the overhead view of the UAV, thereby facilitating consistent feature matching across the two datasets.

By applying the IPM to the MMS data, we converted the street-view MMS images into a top-down perspective that matched the UAV imagery. This transformation allows for direct comparison and feature registration, removing most of the perspective distortion present in the ground-level capture. Once the MMS image was projected onto a top-down view, we used LightGlue when performing feature matching to identify the key points that serve as GCPs to accomplish accurate registration of the UAV and MMS datasets.

**Figure 8:**
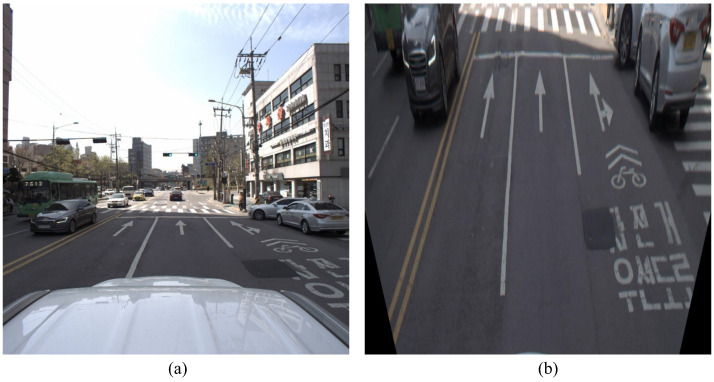
Application of IPM. MMS street-view before (**a**,**b**) after IPM application.

Therefore, IPM is a preparatory step to ensure the compatibility of views emanating from very different perspectives. In conjunction with feature matching, it constitutes a sound basis for the generation of seamless and high-resolution 3D urban maps. Figure 8 shows the results obtained after applying the IPM method.

#### 2.3.5. Matching Points Between UAV Image and MMS Top-View Image

Feature matching between these datasets was performed using LightGlue [43], which is critical for obtaining correctly matched 3D maps. LightGlue introduced deep learning and attention mechanisms to solve complicated problems in feature alignment across large-scale heterogeneous datasets. This approach effectively captures intricate information and aligns features even with significant variations in viewpoint, scale, and lighting conditions.

Matching was performed between the UAV images and two primary MMS data formats: a top-down 2D image derived from the MMS point cloud and MMS street-view images, which were converted to a top-down perspective using IPM. The 2D top-down image generated from the MMS point cloud serves as an overhead view that complements the UAV’s aerial perspective, whereas the IPM transforms the MMS street-view images to align spatially with the UAV imagery, facilitating accurate cross-view feature matching. This dual approach allows LightGlue to bridge aerial and street-level data, creating a unified spatial framework.

LightGlue employs an attention-based mechanism to prioritize high-confidence matches, reducing errors caused by significant differences in perspective. Let $F_{U}$ and $F_{M}$ represent the feature sets extracted from the UAV image and MMS top-view image, respectively. The matching process is formulated as Equation (17):

(17) $$S\left(i,j\right)=\mathrm{softmax}\left(Q_{i}K_{j}^{⊤}\right)\cdot V_{j}$$

where S(i,j) is the similarity score between features *i* and *j*, $Q_{i}$, $K_{j}$, and $V_{j}$ denote the query, key, and value matrices for the features, respectively. These matrices are learned during LightGlue’s training phase to maximize the robustness of feature alignment.

The optimization process minimizes the matching loss *L*, defined as Equation (18):

(18) $$L=\sum_{(i,j)\in M}{∥F_{U}^{i}-F_{M}^{j}∥}^{2}$$

where M represents the set of matched features. This loss function ensures that feature pairs with high similarity scores are closely aligned, improving matching accuracy.

Moreover, LightGlue integrates an outlier rejection mechanism, which uses confidence thresholds to filter mismatched features. Matched features exceeding a predefined confidence threshold τ are selected as GCPs for further integration as in Equation (19):

(19) $$M_{\mathrm{selected}}=\left\{\left(i,j\right)\in M:S\left(i,j\right)>\tau \right\}$$

These selected matches are then incorporated into the SfM pipeline, refining both the EOPs and IOPs.

In many cases, traditional methods for feature matching, such as SIFT, cannot handle the variability between aerial- and ground-based data. However, LightGlue employs a sophisticated training pipeline that can learn complex patterns across perspectives, yielding highly improved accuracy in scene-matching results when there are large-scale differences and viewpoint changes. This adaptiveness is necessary for matching the top-down views from UAVs with the overhead and street-level views of the MMS data to guarantee precision, even in the most challenging urban environments.

Moreover, LightGlue enhances computational efficiency by reducing redundant matching calculations, which is of significant benefit to the high-resolution datasets that come with most UAV and MMS projects. It minimizes computational overhead and optimizes processes without compromising the quality of integration between both sources.

**Figure 9:**
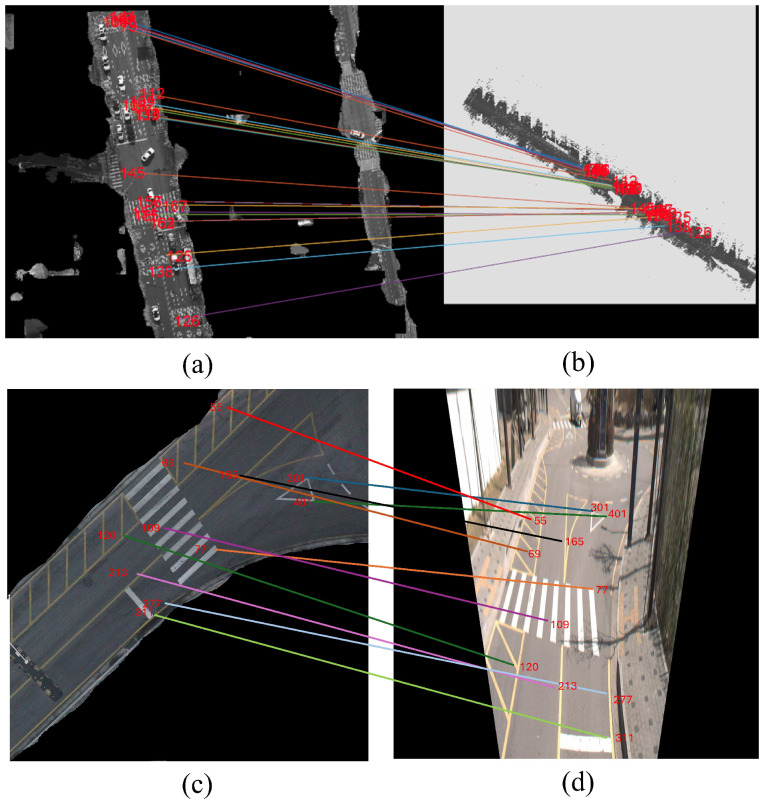
Matched features: (**a**) UAV image after road segmentation, (**b**) MMS top view converted to a 2D image, (**c**) UAV image after road segmentation, and (**d**) street view MMS image.

From this matching framework, we achieved very high feature alignment accuracies for both the UAV and MMS datasets by maintaining matching features with the highest confidence level. This refined feature matching helps complete and obtain an accurate 3D representation, increasing the detail and reliability of the urban map for applications such as road extraction, infrastructure analysis, and broader geospatial studies. Figure 9 presents the matched features between the UAV image, the 2D image from the point cloud, and the street-view image from the MMS.

## 3. Results

### 3.1. Point Cloud Registration and Filtering

GCPs, which were identified and applied to UAV images, MMS point clouds, and street-view images, facilitated the creation of an accurate high-resolution point cloud. This was particularly instrumental in the SfM process, wherein these GCPs provided critical spatial references, anchoring each UAV image and MMS point within a unified 3D coordinate system. Using these GCPs in SfM, we derived a dense and consistent point cloud that captured seamless details for every possible aspect of the study area.

Following the combination of UAV imagery and MMS data into a point cloud, the remaining key challenge is to align these two datasets within one integrated model. With this variability in viewpoints and their respective resolutions, it is necessary to find a reliable registration method to bring them together. To this end, the ICP algorithm, which is one of the most robust methods for aligning point clouds, was adopted. The MMS point cloud had the highest accuracy; therefore, it was assumed to be the master during the registration process. This reference framework of the MMS data allowed us to anchor accurately the UAV-derived point cloud to a stable foundation, minimizing spatial distortions.

In the ICP process, the UAV point cloud is iteratively transformed to align with the MMS image master dataset. Each iteration included changes in the position and orientation of the UAV point cloud, a comparison of points, and a refined alignment based on the nearest points in the MMS dataset. Through the continuous minimization of alignment errors between these two datasets, the ICP achieved near-seamless integration, where the UAV point cloud was fitted closely to the framework of the MMS. This iterative process reduces considerably positional discrepancies and ensures a high degree of coherence between the two data sources. The final dataset combined details from both the top-down (UAV) and side-view (MMS) perspectives.

**Figure 10:**
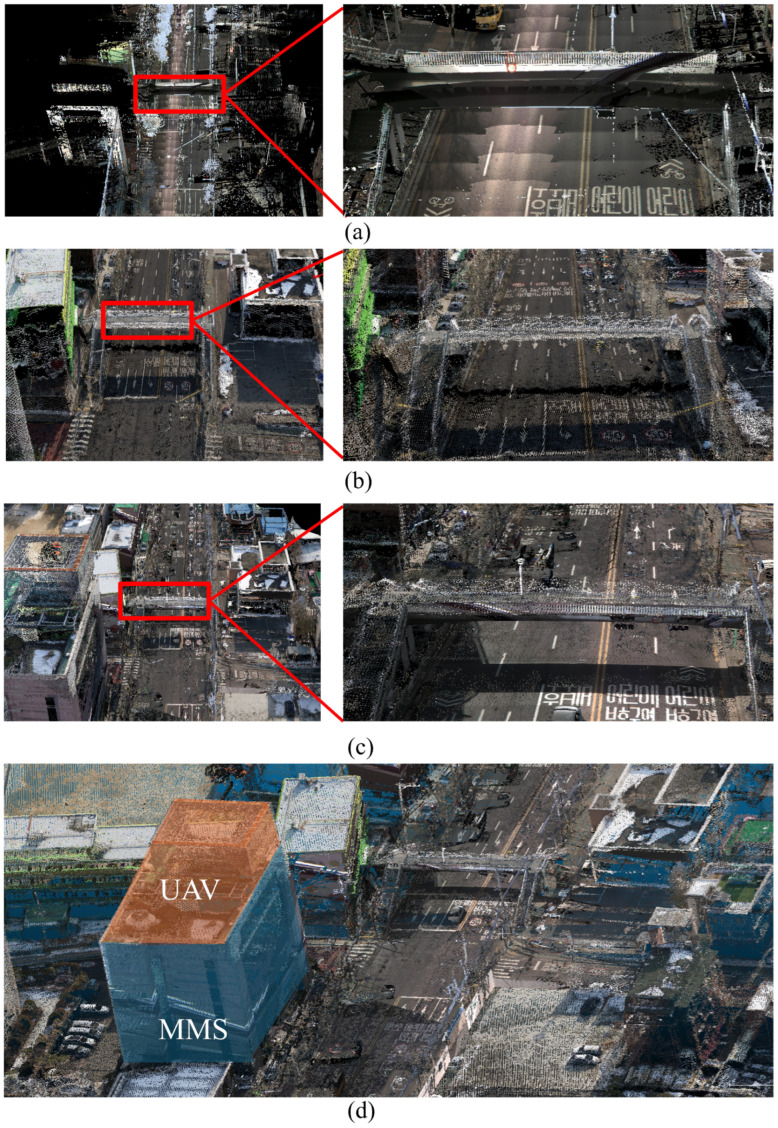
(**a**) Calibrated MMS point cloud, (**b**) UAV-generated point cloud utilizing MMS-extracted GCPs, and (**c**,**d**) integrated point cloud.

After ICP-based alignment, the integrated dataset was further refined using a filtering process. During the filtering process, any noise or outliers introduced into the point cloud during capture or registration were removed. Points that deviated from the master dataset were segregated and excluded from the model to maintain clarity and consistency in the spatial arrangement. The integrated dataset unites the best of both sources: a spatially accurate, coherent, and visually detailed model that serves the purpose of precise mapping, analysis, and visualization. By carefully aligning and filtering the resulting 3D map, the model was of high quality for advanced geospatial analysis, making it highly useful for applications ranging from road extraction to environmental monitoring within the study area. Figure 10 shows the integrated data from the UAV imagery and the calibrated MMS point cloud.

### 3.2. Validation

A comprehensive validation was carried out with an emphasis on completeness and accuracy, two crucial components of 3D urban map production, in order to assess the robustness and application of the proposed method. In order to ensure complete coverage, density analysis, occlusion handling, and visual inspection were used to evaluate the map’s completeness, which measures the inclusion of all key urban features. Accuracy measures the geometric precision of the reconstructed elements by comparing them to reference data, highlighting the effectiveness of the integration framework in achieving spatial fidelity. Together, these validations provide a comprehensive assessment of the methodology’s ability to produce reliable and high-quality urban maps.

#### 3.2.1. Completeness Assessment of the 3D Map

To further assess the effectiveness of the suggested approach, the point clouds’ completeness was compared before and after integration. The analysis focused on numerous crucial factors, such as volume density, occlusion handling, and a thorough visual inspection to find any improvements in the overall quality and consistency of the reconstructed point cloud. Volume density was measured to estimate the level of detail collected in the integrated point cloud, and occlusion handling was examined to assess the method’s capacity to fill gaps and improve coverage in obstructed areas. Visual examination was also undertaken to identify qualitative advantages such as smoother transitions, and improved structural coherence, providing insights into the practical application and resilience of the suggested approach.

***The volume density*** of point clouds was employed as a metric to determine completeness. This was determined by counting the number of points within a sphere of a given radius and normalizing the result to the sphere’s volume. For this work, a radius of 0.05 m was used to balance computing efficiency and spatial resolution. Prior to using the proposed method, the volume density obtained just from UAV data was 92.687. After incorporating the MMS dataset, the density increased dramatically to 228.34. Furthermore, the integration of MMS data had a particularly significant influence on road regions, where the MMS system’s high-resolution data collecting was concentrated. This enhancement is due not just to the inherent higher density of the MMS data, but also to the function of GCPs extracted from the MMS dataset in the SfM process. The addition of these GCPs improved the SfM workflow by allowing it to generate a denser, more coherent, and higher-quality point cloud.

**Figure 11:**
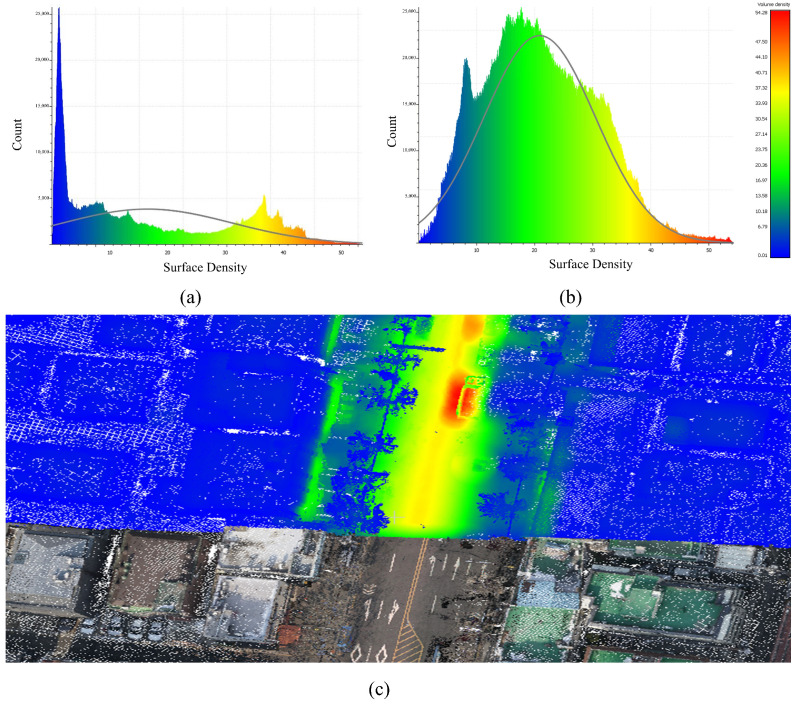
Surface density distribution of point cloud density before (**a**) and after (**b**) integration, with (**c**) showing improved density, especially in road areas with MMS data.

Figure 11 shows the visual representation of the results. Figure 11a shows the distribution of surface density for the point cloud derived purely from UAV data, which shows lower density values due to the restricted resolution of UAV data. Figure 11b illustrates the distribution of surface density following the integration of the MMS dataset, demonstrating a clear trend towards higher density values as a result of the integration procedure. Figure 11c depicts the density improvements, focusing on the increased density in road areas. The enhanced density in these places highlights the MMS dataset’s considerable contribution to improving the overall quality of the integrated point cloud.

***Occlusion handling*** was another essential component of the evaluation. To detect occluded areas, the method mimicked each point in the point cloud’s exposure to surrounding geometry by casting numerous rays from each point and calculating how much of the environment was visible. Points with poor visibility were classified as obscured or recessed. To obtain precise and detailed findings, the simulation took into account aspects such as ray number and resolution. Each point was assigned a value representing its exposure to ambient visibility. Points in darker sections corresponded to areas with greater occlusion.

Illuminance distributions were further examined to assess the reduction in occlusion. Prior to using the proposed approach, the average illuminance was 0.374 with a standard variation of 0.213. The low mean value suggests that major areas of the dataset are poorly illuminated or obscured, but the high standard deviation indicates that lighting conditions are uneven and that illuminance varies significantly across the dataset. After processing, the mean illuminance increased to 0.429 while the standard deviation fell to 0.189. The greater mean indicates that more points in the dataset are well-illuminated, with previously obscured regions becoming more visible as a result of MMS data integration. Meanwhile, the lower standard deviation indicates a more stable and uniform illuminance distribution, with less variation in lighting circumstances.

**Figure 12:**
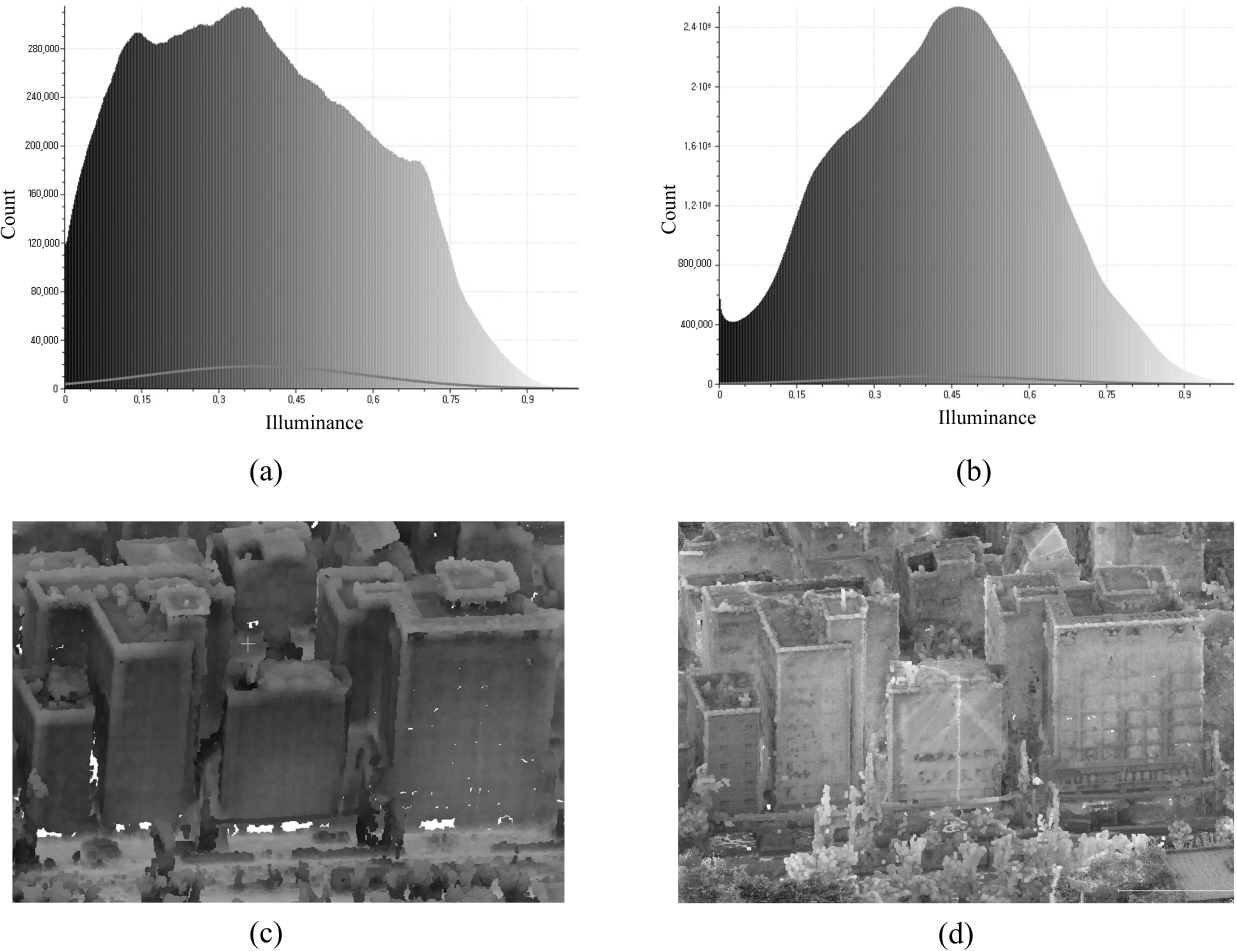
(**a**) Illuminance distribution of the UAV-generated point cloud, (**b**) illuminance distribution of the integrated point cloud, (**c**,**d**) showcases of illumination for the UAV-generated and integrated point clouds, respectively.

Because the UAV-generated point cloud covers the same area in both datasets, the observed improvements can be attributed to the inclusion of MMS data, which effectively filled in occluded parts, reduced variability, and improved overall lighting. These results show that the proposed strategy successfully reduced occlusions while improving the dataset’s completeness, quality, and uniformity. Figure 12 depicts the illuminance distribution histograms of UAV-generated and integrated point clouds, highlighting the overall changes in illuminance before and after processing. Figure 12c,d use the same building to demonstrate the variations in illumination. The UAV-generated point cloud in Figure 12c has darker parts and uneven illumination due to severe obstruction and low vision. In comparison, the integrated point cloud in Figure 12d is brighter and more evenly illuminated, demonstrating that MMS data integration significantly reduced obstructed regions, improved visibility, and increased the dataset’s completeness and uniformity.

**Figure 13:**
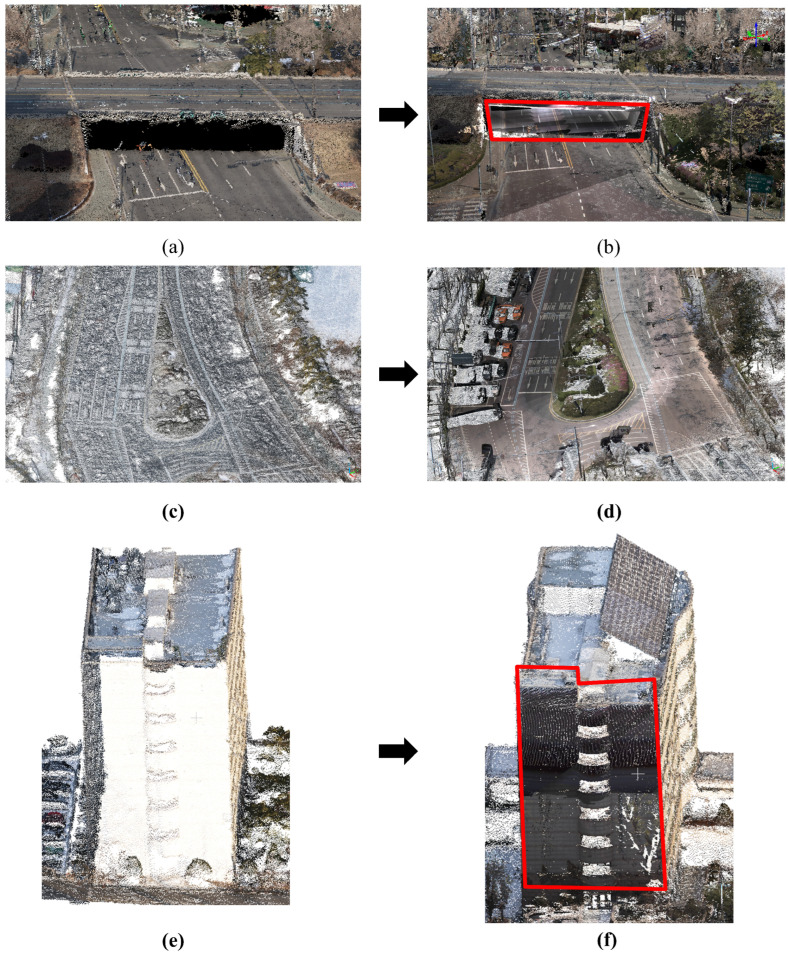
Enhanced point clouds combining UAV and MMS data: (**a**,**b**) underpass, (**c**,**d**) street, (**e**,**f**) building façade.

The original and integrated datasets were overlayed to conduct a ***Qualitative visual assessment*** of the point clouds. This approach allowed for a recognition of enhancements in detail and spatial coherence. Smoother transitions, clearer definitions of structural parts, and more full representations of urban features were all seen in the combined dataset. Notably, the integration of UAV and MMS data increased the completeness of places such as roads and underpasses, as well as the building façade near roadways, where MMS data are highly accurate. Figure 13 shows the improvements: Figure 13a,b exhibit the underpass case, Figure 13c,d depict the road case, and Figure 13e,f show the building façade case. The red boxes in the photos represent MMS areas added to the low-cost UAV-generated point cloud, highlighting the suggested method’s improved detail and spatial coverage.

The combination of volume density analysis, occlusion handling, and visual inspection highlights the robustness of the proposed framework in enhancing point cloud completeness. By improving point cloud density, reducing occlusions, and achieving visual coherence, the method ensures the production of high-quality datasets that are critical for urban planning, disaster response, and infrastructure monitoring.

#### 3.2.2. Accuracy Assessment of the 3D Map

Our integrated 3D model, produced through the fusion of low-cost UAV and MMS data, was validated to identify its spatial accuracy. To this end, the GCPs and independent checkpoints spread over the study area were considered to check their precision in three different configurations: no GCPs, GNSS-based GCPs, and MMS-extracted GCPs. This was carried out so that the effect of each GCP source on geolocation accuracy and model consistency could be measured.

Forty points were collected using the highly accurate positioning capability of GNSS with the VRTK technique. Sampling was undertaken in the study area of Yonsei University and the Yeonhui-dong neighborhood to obtain a representative sample of the spatial variability in this urban environment. Among these, 20 were GCPs, which were helpful in the alignment of the model, whereas the remaining 20 served as independent checkpoints to evaluate the accuracy of the model in areas that were not directly influenced by GCPs.

For validation, three configurations of the model were compared: without GCPs, with GCPs based on GNSS, and with GCPs extracted from the MMS. The first configuration did not consider any GCPs and was treated as a baseline to establish the inherent accuracy of the alignment between the UAV and MMS datasets without external references. In the second configuration, GNSS-based GCPs were considered to investigate the improvement in the alignment accuracy owing to these high-precision points. Finally, the GCPs for the third configuration were derived from the MMS features, which were expected to achieve the best georeferencing alignment because of their close correlation with the spatial framework of the MMS dataset.

**Figure 14:**
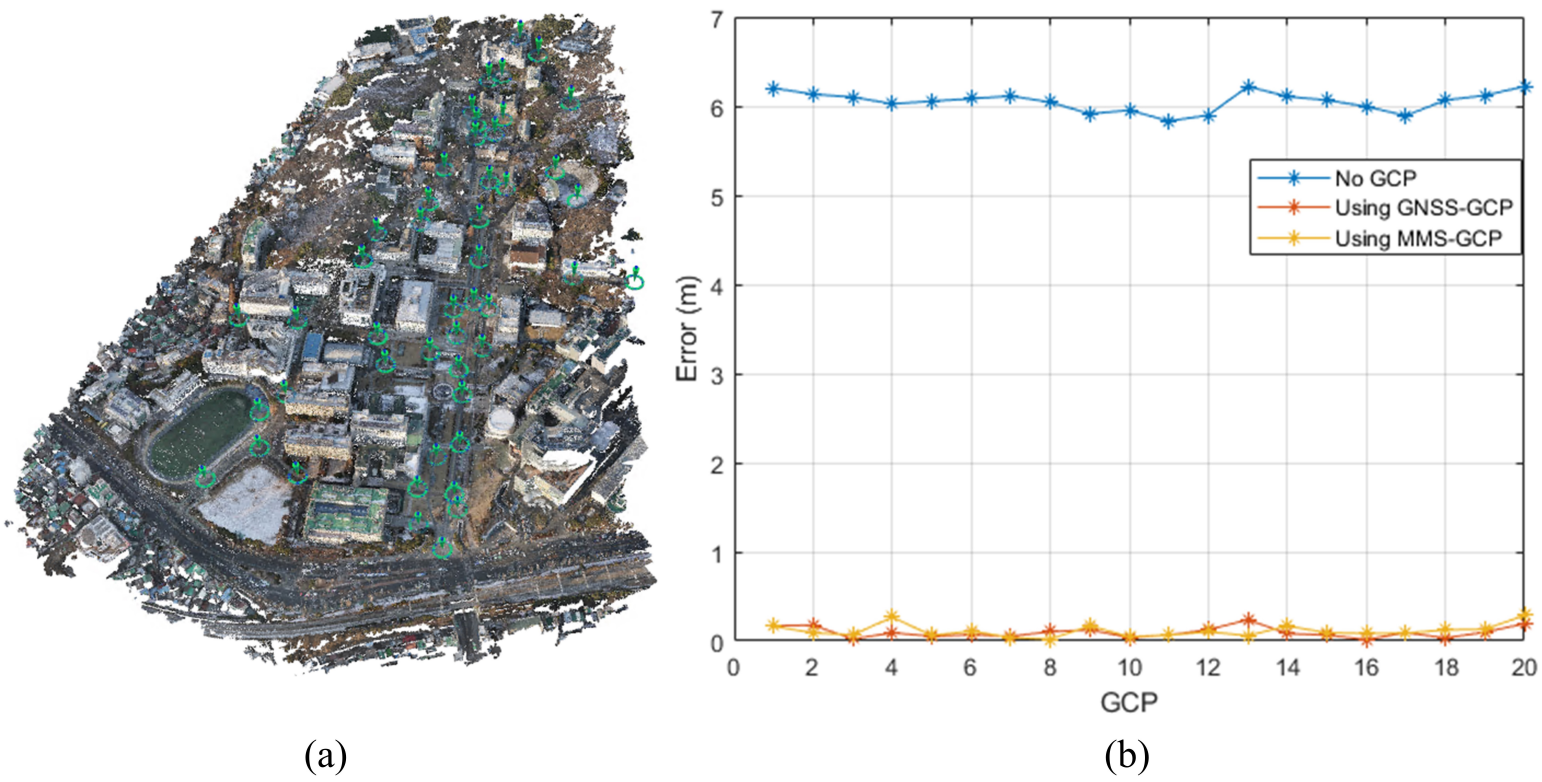
(**a**) GCP locations from GNSS, (**b**) Geolocation accuracy comparison.

Figure 14 shows the geolocation accuracy of the three configurations. The results suggest that the configuration using GNSS-based GCPs yielded the lowest RMSE, followed closely by the configuration that used MMS-extracted GCPs. The RMSE values for X, Y, and Z for each configuration are summarized in Table 3. We can also see that the MMS-GCP configuration had the highest inaccuracy with an RMSE of 0.1311 m, whereas the GNSS-GCP configuration yielded an RMSE of 0.1121 m. In contrast, the basic configuration without GCPs yielded an RMSE of 6.0528 m, indicating that the presence of GCPs, particularly those that have been extracted from the MMS data, increases considerably the attained accuracy.

**Figure 15:**
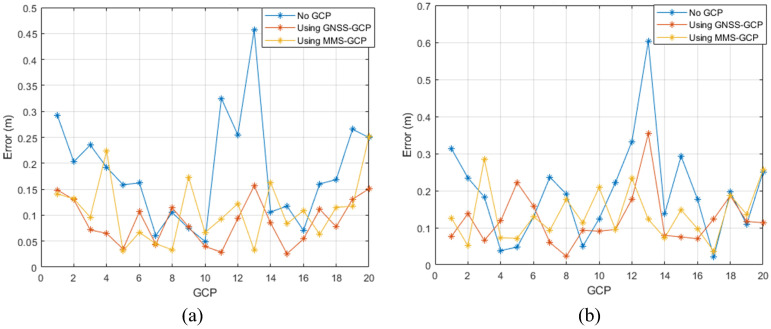
(**a**) Residual of 3D registration, (**b**) Independent check results.

To investigate further the precision of their alignment, we conducted a residual analysis from the 3D registration process as well as for the independent checkpoints. By performing a residual analysis, we determined the spatial consistency of each configuration. Figure 15 depicts the residuals of each configuration. Separate charts were used for the 3D registration residuals and independent checkpoint residuals. A detailed breakdown of the residuals for each configuration is presented in Table 4.

Regarding the 3D registration residuals and independent checkpoint residuals, the minimum error values belonged to the MMS-GCP configuration, indicating that the integration of MMS-derived GCPs yielded the highest spatial alignment accuracy. In the independent checkpoint residuals, the MMS-GCP configuration yielded results nearly identical to those of the GNSS-GCP setup, demonstrating the model’s robustness and spatial accuracy.

These results confirm that the use of MMS-extracted GCPs increases considerably the spatial accuracy and consistency of data alignment. The MMS GCP setup minimized the residuals in 3D registration with the lowest errors in the independent checkpoints, thus confirming the high accuracy and reliability of the integrated 3D data obtained using MMS-based GCPs. The validation underlines the novelty of the use of MMS data as GCPs, increasing the inner spatial coherence of 3D data, and allowing its further employment in more challenging geospatial tasks above urban areas.

## 4. Discussion

This study presented a detailed framework for the generation of highly accurate 3D urban maps that merge low-cost UAV imagery and calibrated MMS point cloud datasets, each of which is crucial to the attained accuracies. It combined advanced techniques of feature extraction, transformation, and alignment, including U²-net-based road segmentation, CSF, IPM, and feature matching using LightGlue. The framework yielded an RMSE of 0.131 m, which not only met but also outperformed recent benchmarks, such as those in [17,24]. The structured integration of these diverse methods enabled the development of a coherent model such that no single step accounted solely for the observed accuracy gains.

It was thus important, to begin with the initial step of road segmentation using U²-Net on UAV imagery in conjunction with CSF to isolate ground-level features in the MMS data and to set a solid, road-based foundation for the alignment. The road features acted as continuous and trustworthy features in both datasets, ensuring that the main urban structures are emphasized in both the UAV and MMS data. Converting the MMS point cloud data into a 2D top-view intensity image and performing IPM on the MMS street-view images allowed both datasets to share a common overhead perspective, simplifying the cross-view comparisons. Feature matching was performed using LightGlue, which further enforced alignment using attention mechanisms that adaptively considered both scale and perspective differences. Unlike many feature-matching approaches, LightGlue effectively handles the variability between data from UAV and MMS, thereby enhancing the robustness of the feature alignment.

Recent advancements in multimodal data fusion provide important insights into how the proposed method contributes to the field. For instance, one study developed a LiDAR-aided refinement strategy for UAV-mounted cameras that achieved centimeter-level accuracy by using LiDAR-derived control points in conjunction with GNSS-acquired GCPs to refine IOPs for SfM [16]. This approach achieves excellent spatial accuracy by integrating LiDAR data with GNSS-supported GCPs and refining the point cloud iteratively. However, its dependency on GNSS and LiDAR systems makes it expensive and impractical in metropolitan areas with poor GNSS coverage. In contrast, the solution we used completely eliminates GNSS dependency by leveraging MMS-derived GCPs, which automates the procedure and achieves comparable alignment precision at a lower cost.

Another method integrates UAV images with terrestrial images to reconstruct urban scenes using SfM [17]. Sparse models are created for both datasets, and cross-platform tie points are refined to align terrestrial and aerial data through a 3D similarity transformation and global optimization. While effective, this method is strongly reliant on GNSS data and computationally demanding refinement, which limits its scalability in metropolitan regions with GNSS restrictions. However, while the pipeline is praised for its dependability in congested urban scenes, the research does not explicitly mention quantitative accuracy measurements, such as RMSE, for integrated reconstruction. The approach we proposed eliminates GNSS dependency by utilizing MMS-derived GCPs, resulting in higher alignment precision in a more feasible and scalable procedure.

Similarly, combining oblique UAV images with MMS point clouds has been demonstrated to improve facade reconstruction and geolocation accuracy [24]. Their method highlighted the significance of vertical GCP distributions and decreased RMSE values to about 16 cm in the X, Y, and Z directions. However, their procedure necessitates the hand extraction of GCPs, which presents operational difficulties. Our proposed framework employs an automated alignment process that minimizes manual intervention, achieving a superior RMSE of 0.131 m while simplifying the implementation. These distinctions underscore the accessibility and efficiency of our proposed methodology.

These distinctions underscore the accessibility and efficiency of our proposed methodology. To improve forest canopy models, UAV images were integrated with TLS data [44]. This method aligns UAV and TLS point clouds using GNSS-acquired GCPs, incorporating canopy and understory data to improve accuracy. However, this method necessitates substantial ground GCP collecting, which is time-consuming and inflexible for urban applications. In contrast, the proposed fusion approach completely eliminates the necessity for ground GCP collecting by utilizing MMS-derived GCPs.

A deep learning-based multimodal fusion network, PMNet, was introduced to fuse LiDAR and aerial imagery for 3D semantic segmentation [45]. While their feature-level fusion strategy demonstrates excellent results in segmentation tasks, it primarily addresses classification accuracy rather than precise geometric integration. Our approach, on the other hand, directly addresses the alignment of geometric and spectral data to produce dense and seamless point clouds, enabling its application in urban 3D modeling and reconstruction.

Efforts to map terrains using UAV image-based point clouds have demonstrated potential but face significant challenges under dense canopy cover, where occlusions obscure ground visibility [46]. By combining images captured at different angles, this approach reduces RMSE errors in complex environments such as dense canopy cover. However, terrain mapping approaches that rely entirely on UAV imagery struggle to attain the necessary completeness for urban applications. Our proposed approach addresses these challenges by combining nadir UAV imagery and MMS point clouds, resulting in improved coverage and alignment precision for both façades and terrain in urban contexts.

Fusing LiDAR data with image-based point clouds has been shown to generate dense 3D models [47]. These systems rely on thorough calibration processes and manual tie-point extraction for integration, which can produce precise results depending on input quality. However, such technologies frequently require substantial manual labor, limiting their scalability in large-scale applications or complex urban settings.

Residual analyses at independent checkpoints demonstrated the accuracy and robustness of the model. The small residual values, particularly from the configurations with MMS-derived GCPs, proved that spatial coherence was maintained outside the affected areas of the GCPs. This result confirms the reliability of GCPs derived from MMS as an efficient alternative to those obtained from GNSS, especially for urban areas characterized by limited accessibility to GNSS. These minimized residuals further strengthen the potential of the framework for applications in domains requiring high spatial fidelity, such as urban planning, infrastructure monitoring, and emergency responses.

In a broader context, the findings suggest that adaptive feature matching, consistent perspective translation, focused road feature extraction, and a suitable UAV-MMS fusion strategy could be a reliable method for comprehensive urban mapping. Furthermore, in order to facilitate the creation of more intricate characteristics, future studies could improve this framework by adding new data sources, including oblique images or Internet-of-Things (IoT) sensors. Expanding upon this, future studies will explore the inclusion of datasets from diverse geographic and environmental contexts to evaluate the robustness and scalability of the proposed method. This approach will ensure the framework’s adaptability to varied urban and rural settings, providing a broader validation of its precision and versatility.

## 5. Conclusions

This study presents a stable and scalable system for producing seamless, high-fidelity 3D metropolitan maps by combining low-cost UAV footage with calibrated MMS point cloud data. The suggested strategy utilizes sophisticated techniques such as U²-Net-based road segmentation, CSF, IPM, and LightGlue-based feature matching to overcome the constraints of single-source mapping. By integrating these methods into a unified workflow, the framework provides high-resolution spatial modeling with a validated accuracy of 0.131 m, exceeding comparable field methods.

The results show significant increases in completeness and spatial accuracy, as shown by higher point cloud density and better occlusion handling. For example, integrating UAV and MMS information enhanced point cloud volume density in crucial locations like roadways from 92.687 to 228.34. Illuminance measurements confirmed reduced occlusions and increased uniformity, demonstrating the integrated model’s robustness in urban mapping environments. These advances are especially important for applications requiring precise geographic data, such as urban planning, disaster management, and infrastructure monitoring.

Notably, using MMS-derived GCPs as an alternative to traditional GNSS-based techniques improves the proposed framework’s practicality and cost-effectiveness, particularly in metropolitan areas with restricted GNSS access. Automated and hierarchical integration of aerial and ground-level datasets reduces the need for manual intervention while preserving high accuracy and spatial coherence.

Future research could expand on this approach to include new data sources, such as oblique images or IoT sensor data, to improve the spatial and contextual detail of 3D models. Furthermore, the use of real-time processing techniques and increased automation in feature matching and GCP extraction may broaden the methodology’s applicability to dynamic and large-scale urban mapping projects.

In conclusion, the proposed framework establishes a new standard for cost-effective and accurate 3D urban mapping, providing significant improvements over previous techniques. Its scalability, resilience, and adaptability make it a potential solution for a variety of geospatial applications, highlighting its importance in furthering urban modeling and analysis.

## Figures and Tables

**Table 1:** WingtraOne specifications.

Specification	Details	Additional Notes
max flight time	up to 59 min per battery charge	-
flight speed	16 m/s (57.6 km/h)	efficient area coverage
coverage	maps up to 400 hectares per flight at 1 cm/pixel GSD	high-resolution over large areas
weight	3.7 kg, including battery and pay-load	portable for field conditions
camera payload	Sony RX1R II	42 MPixel resolution 7952 × 5304 pixels

**Table 2:** LiDAR system specifications.

Specification	Details	Additional Notes
number of LiDAR sensors	dual LiDAR sensors for enhanced depth and density	improved spatial accuracy
maximum scan rate	up to 1 million points per second	rapid, dense point cloud generation
field of view (FOV)	horizontal FOV of 360°	comprehensive capture of surroundings
camera configuration	multiple RGB cameras for full-color 3D point cloud with 360° overlay	high-quality visualization
GNSS and IMU Integration	high-precision GNSS/IMU system	centimeter-level georeferencing accuracy
data output	RGB, intensity, and geospatial data (GNSS coordinates) per point	detailed point information

**Table 3:** Geolocation accuracy for different GCP configurations (in m).

Configuration	X Error	Y Error	Z Error	RMSE
without GCP	1.6867	6.0355	8.4045	6.0528
GNSS-GCP	0.1268	0.1165	0.0896	0.1121
MMS-GCP	0.1261	0.1768	0.0664	0.1311

**Table 4:** Residuals of 3D registration and independent check points (in m).

Configuration	3D Registration Residuals	Independent Check Residuals
without GCP	0.2307, 0.2587, 0.1147, 0.2108	0.1927, 0.3211, 0.1525, 0.2334
GNSS-GCP	0.1062, 0.1057, 0.0743, 0.0965	0.1459, 0.1757, 0.0862, 0.1409
MMS-GCP	0.1336, 0.1575, 0.0539, 0.1232	0.1685, 0.1826, 0.0850, 0.1516

## Data Availability

The data are not publicly available because they are only accessible to Korean citizens owing to legal restrictions.
